# Nonexistence of stable *F*-stationary maps of a functional related to pullback metrics

**DOI:** 10.1186/s13660-017-1483-z

**Published:** 2017-09-08

**Authors:** Jing Li, Fang Liu, Peibiao Zhao

**Affiliations:** Department of Applied Mathematics, Nanjing University of Science and Technology, Nanjing, Jiangsu 210094 P.R. China

**Keywords:** 58E20, 53C21, *F*-stationary map, compact convex hypersurfaces

## Abstract

Let $M^{m}$ be a compact convex hypersurface in $R^{m+1}$. In this paper, we prove that if the principal curvatures $\lambda_{i}$ of $M^{m}$ satisfy $0<\lambda_{1}\leq \cdots \leq \lambda_{m}$ and $3\lambda_{m}<\sum_{j=1}^{m-1}\lambda_{j}$, then there exists no nonconstant stable *F*-stationary map between *M* and a compact Riemannian manifold when (6) or (7) holds.

## Introduction

Let $u:(M^{m},g)\rightarrow (N^{n},h)$ be a smooth map between Riemannian manifolds $(M^{m},g)$ and $(N^{n},h)$. Recently, Kawai and Nakauchi [1] introduced a functional related to the pullback metric $u^{*}h$ as follows:

1 $$\begin{aligned} \Phi (u)=\frac{1}{4} \int_{M} \bigl\Vert u^{*}h \bigr\Vert ^{2}\,dv_{g}, \end{aligned}$$

(see [2–4]), where $u^{*}h$ is the symmetric 2-tensor defined by

$$\begin{aligned} \bigl(u^{*}h \bigr) (X,Y)=h \bigl(du(X),du(Y) \bigr) \end{aligned}$$

for any vector fields *X*, *Y* on *M* and $\Vert u^{*}h \Vert $ is given by

$$\begin{aligned} \bigl\Vert u^{*}h \bigr\Vert ^{2}=\sum _{i,j=1}^{m} \bigl[h \bigl(du(e_{i}),du(e_{j}) \bigr) \bigr]^{2}, \end{aligned}$$

with respect to a local orthonormal frame $(e_{1},\ldots ,e_{m})$ on $(M,g)$. The map *u* is stationary for Φ if it is a critical point of $\Phi (u)$ with respect to any compact supported variation of *u*, and *u* is stable if the second variation for the functional $\Phi (u)$ is nonnegative. They showed the nonexistence of a nonconstant stable stationary map for Φ, either from $S^{m}$ ($m\geq 5$) to any manifold, or from any compact Riemannian manifold to $S^{n}$ ($n\geq 5$). In this paper, for a smooth function $F:[0,\infty )\rightarrow [0,\infty )$ such that $F(0)=0$ and $F'(t)>0$ on $t\in (0,\infty )$, we are concerned with the instability of *F*-stationary maps which is the generalization of a stationary map for Φ introduced by Asserda in [4]. In [4], they obtained some monotonicity formulas for *F*-stationary maps via the coarea formula and the comparison theorem. Also, by using monotonicity formulas, they got some Liouville type results for these maps.

The authors in [5] obtained the first and second variation formula for *F*-stationary maps. By using the second variation formula, they proved that every stable *F*-stationary map from $S^{m}(1)$ to any Riemannian manifold is constant if

2 $$\begin{aligned} \int_{S^{m}} \bigl\Vert u^{*}h \bigr\Vert ^{2} \biggl\{ F'' \biggl(\frac{\Vert u^{*}h \Vert ^{2}}{4} \biggr) \bigl\Vert u^{*}h \bigr\Vert ^{2}+(4-m)F' \biggl(\frac{\Vert u^{*}h \Vert ^{2}}{4} \biggr) \biggr\} \,dv_{g}< 0, \end{aligned}$$

or every *F*-stationary map from any compact Riemannian manifold $N^{n}$ to $S^{m}$ is constant if

3 $$\begin{aligned} \int_{N^{n}} \bigl\Vert u^{*}h \bigr\Vert ^{2} \biggl\{ F'' \biggl(\frac{\Vert u^{*}h \Vert ^{2}}{4} \biggr) \bigl\Vert u^{*}h \bigr\Vert ^{2}+(4-m)F' \biggl(\frac{\Vert u^{*}h \Vert ^{2}}{4} \biggr) \biggr\} \,dv_{g}< 0. \end{aligned}$$

In this paper, we obtain the results on the instability of *F*-stationary maps which are from or into the compact convex hypersurfaces in the Euclidean space.

## Preliminaries

Let $F:[0,\infty )\rightarrow [0,\infty )$ be a $C^{2}$-function such that $F(0)=0$ and $F'(t)>0$ on $t\in (0,\infty )$. For a smooth map $u:(M,g)\rightarrow (N,h)$ between compact Riemannian manifolds $(M,g)$ and $(N,h)$ with Riemannian metrics *g* and *h*, respectively, following Ara [6] for an *F*-harmonic map (also see [7–10]), Asserda in [4] gave the following definition.

### Definition 2.1

We call *u* an *F*-stationary map for $\Phi_{F}$ if

$$ \frac{d}{dt}\Phi_{F}(u_{t})|_{t=0}=0 $$

for any compactly supported variation $u_{t}:M\rightarrow N$ with $u_{0}=u$, where

$$ \Phi_{F}(u)= \int_{M^{m}}F \biggl(\frac{\Vert u^{*}h \Vert ^{2}}{4} \biggr)\,dv_{g}. $$

Let ∇ and ^*N*^∇ always denote the Levi-Civita connections of *M* and *N*, respectively. Let ∇̃ be the induced connection on $u^{-1}TN$ defined by $\widetilde{\nabla } _{X}W=^{N}\nabla_{du(X)}W$, where *X* is a tangent vector of *M* and *W* is a section of $u^{-1}TN$. We choose a local orthonormal frame field $\{e_{i}\}$ on *M*. We define the *F*-tension field $\tau_{\Phi_{F}}(u)$ of *u* by

4 $$\begin{aligned} \tau_{\Phi_{F}}(u) =&-\delta \biggl(F' \biggl( \frac{\Vert u^{*}h \Vert ^{2}}{4} \biggr)\sigma_{u} \biggr) \\ =&F' \biggl(\frac{\Vert u^{*}h \Vert ^{2}}{4} \biggr)\operatorname{div}_{g}( \sigma_{u})+\sigma_{u} \biggl(\operatorname{grad} \biggl(F' \biggl(\frac{\Vert u^{*}h \Vert ^{2}}{4} \biggr) \biggr) \biggr), \end{aligned}$$

where $\sigma_{u}=\sum_{j}h(du(\cdot ),du(e_{j}))du(e_{j})$, which was defined in [1].

We need the following second variation formula for *F*-stationary maps (cf. [5]). Let $u:(M,g)\rightarrow (N,h)$ be an *F*-stationary map. Let $u_{s,t}:M\rightarrow N$ ($-\varepsilon < s,t< \varepsilon $) be a compactly supported two-parameter variation such that $u_{0,0}=u$, and set $V=\frac{\partial }{\partial t}u_{s,t}|_{s,t=0}$, $W=\frac{\partial }{ \partial s}u_{s,t}|_{s,t=0}$. Then

$$\begin{aligned} \frac{\partial^{2}}{\partial s\partial t}\Phi_{F}(u_{s,t})|_{s,t=0} &= \int_{M}F'' \biggl(\frac{\Vert u^{*}h \Vert ^{2}}{4} \biggr)\langle \widetilde{\nabla }V,\sigma_{u}\rangle \langle \widetilde{\nabla }W,\sigma_{u}\rangle \,dv_{g} \\ &\quad {}+ \int_{M}F' \biggl(\frac{\Vert u^{*}h \Vert ^{2}}{4} \biggr)\sum _{i,j=1}^{m}h( \widetilde{\nabla }_{e_{i}}V,\widetilde{\nabla }_{e_{j}}W)h \bigl(du(e_{i}),du(e _{j}) \bigr)\,dv_{g} \\ &\quad {}+ \int_{M}F' \biggl(\frac{\Vert u^{*}h \Vert ^{2}}{4} \biggr)\sum _{i,j=1}^{m}h \bigl( \widetilde{\nabla }_{e_{i}}V,du(e_{j}) \bigr)h \bigl(\widetilde{\nabla }_{e_{i}}W,du(e _{j}) \bigr)\,dv_{g} \\ &\quad {}+ \int_{M}F' \biggl(\frac{\Vert u^{*}h \Vert ^{2}}{4} \biggr)\sum _{i,j=1}^{m}h \bigl( \widetilde{\nabla }_{e_{i}}V,du(e_{j}) \bigr)h \bigl(du(e_{i}), \widetilde{\nabla }_{e_{j}}W \bigr)\,dv_{g} \\ &\quad {}+ \int_{M}F' \biggl(\frac{\Vert u^{*}h \Vert ^{2}}{4} \biggr)h \bigl(R^{N} \bigl(V,du(e_{i}) \bigr)W,du(e_{j}) \bigr)h \bigl(du(e _{i}),du(e_{j}) \bigr) \,dv_{g}, \end{aligned}$$

where $\langle \cdot, \cdot \rangle $ is the inner product on $T^{*}M\otimes u^{-1}TN$ and $R^{N}$ is the curvature tensor of *N*.

We put

5 $$\begin{aligned} I(V,W)=\frac{\partial^{2}}{\partial s\partial t}\Phi_{F}(u_{s,t})|_{s,t=0}. \end{aligned}$$

An *F*-stationary map *u* is called stable if $I(V,V)\geq 0$ for any compactly supported vector field *V* along *u*.

## *F*-stationary maps from compact convex hypersurfaces

In this section, we obtain the following result.

### Theorem 3.1

*Let* $M\subset R^{m+1}$ *be a compact convex hypersurface*. *Assume that the principal curvatures* $\lambda_{i}$ *of* $M^{m}$ *satisfy* $0<\lambda_{1} \leq \cdots \leq \lambda_{m}$ *and* $3\lambda_{m}< \sum_{i=1}^{m-1} \lambda_{i}$. *Then every nonconstant* *F*-*stationary map from* *M* *to any compact Riemannian manifold* *N* *is unstable if there exists a constant* $c_{F}=\operatorname{inf}\{c\geq 0| F'(t)/t^{c}\ \textit{is nonincreasing}\}$ *such that*

6 $$\begin{aligned} c_{F}< \frac{1}{4\lambda^{2}_{m}} \min_{1\leq i\leq m} \Biggl\{ \lambda_{i} \Biggl( \sum_{k=1}^{m} \lambda_{k}-2\lambda_{i}-2\lambda_{m} \Biggr) \Biggr\} \end{aligned}$$

*or when* $F''(t)=F'(t)$ (*for example*, $F(t)=\exp (t)$)

7 $$\begin{aligned} \bigl\Vert u^{*}h \bigr\Vert ^{2}< \frac{1}{\lambda_{m}^{2}} \min_{1\leq i\leq m} \Biggl\{ \lambda _{i} \Biggl(\sum_{k=1}^{m}\lambda_{k}-2 \lambda_{i}-2\lambda_{m} \Biggr) \Biggr\} . \end{aligned}$$

### Proof

In order to prove the instability of $u:M^{m}\rightarrow N$, we need to consider some special variational vector fields along *u*. To do this, we choose an orthonormal field $\{e_{i},e_{m+1}\}$, $i=1,\ldots ,m$, of $R^{m+1}$ such that $\{e_{i}\}$ are tangent to $M^{m}\subset R^{m+1}$, $e_{m+1}$ is normal to $M^{m}$ and $\nabla_{e_{i}}e_{j}|_{P}=0$. Meanwhile, we take a fixed orthonormal basis $E_{A}$, $A=1,\ldots ,m+1$, of $R^{m+1}$ and set

8 $$\begin{aligned} V_{A}=\sum_{i=1}^{m}v_{A}^{i}e_{i}, \quad v_{A}^{i}=\langle E_{A},e_{i} \rangle, v_{A}^{m+1}=\langle E_{A},e_{m+1} \rangle, \end{aligned}$$

where $\langle \cdot, \cdot \rangle $ denotes the canonical Euclidean inner product. Then $du(V_{A})\in \Gamma (u^{-1}TN)$ and

9 $$\begin{aligned}& \sum_{A}v_{A}^{i}v_{A}^{j} = \sum_{A}\langle E_{A},e_{i} \rangle \langle E_{A},e_{j}\rangle= \delta_{ij}, \end{aligned}$$

10 $$\begin{aligned}& \nabla_{e_{i}}V_{A}=v_{A}^{m+1}B_{ij}e_{j}, \end{aligned}$$

11 $$\begin{aligned}& \nabla_{e_{i}}(\nabla_{e_{i}}V_{A}) = -v_{A}^{k}B_{ik}B_{ij}e_{j}+v _{A}^{m+1}(\nabla_{e_{i}}h_{ij})e_{j}, \end{aligned}$$

12 $$\begin{aligned}& \widetilde{\nabla }_{e_{i}} \bigl(du(\nabla_{e_{i}}V_{A}) \bigr) = -v_{A}^{k}B _{ik}B_{ij} du(e_{j}) \\& \hphantom{\widetilde{\nabla }_{e_{i}} (du(\nabla_{e_{i}}V_{A}) )= }{} +v_{A}^{m+1}(\nabla_{e_{i}}B_{ij}) du(e_{j})+v_{A}^{m+1}B_{ij} \widetilde{ \nabla }_{e_{i}}du(e_{j}), \end{aligned}$$

where $B_{ij}$ denotes the components of the second fundamental form of $M^{m}$ in $R^{m+1}$. Suppose that $u:M^{m}\rightarrow N$ is a nonconstant *F*-stationary map. Then the condition $\tau_{F}(u)=- \delta (F'(\frac{\Vert u^{*}h \Vert ^{2}}{4})\sigma_{u})=0$ implies that

13 $$\begin{aligned}& \sum_{A} \int_{M^{m}}F' \biggl(\frac{\Vert u^{*}h \Vert ^{2}}{4} \biggr) \bigl\langle (\triangle du) (V _{A}),\sigma_{u}(V_{A}) \bigr\rangle \, dv_{g} \\& \quad = \sum_{A} \int_{M^{m}}F' \biggl(\frac{\Vert u^{*}h \Vert ^{2}}{4} \biggr)v_{A}^{i}v^{j}_{A} \bigl\langle ( \triangle du) (e_{i}),\sigma_{u}(e_{j}) \bigr\rangle \, dv_{g} \\& \quad = \sum_{i} \int_{M^{m}}F' \biggl(\frac{\Vert u^{*}h \Vert ^{2}}{4} \biggr) \bigl\langle (\triangle du) (e _{i}),\sigma_{u}(e_{i}) \bigr\rangle \, dv_{g} \\& \quad = \int_{M^{m}} F' \biggl(\frac{\Vert u^{*}h \Vert ^{2}}{4} \biggr) \bigl\langle ( \triangle du),\sigma _{u} \bigr\rangle \,dv_{g} \\& \quad = \int_{M^{m}} \biggl\langle \delta du,\delta \biggl(F' \biggl( \frac{\Vert u^{*}h \Vert ^{2}}{4} \biggr)\sigma _{u} \biggr) \biggr\rangle \,dv_{g} =0. \end{aligned}$$

It follows from the Weitzenböck formula that

14 $$\begin{aligned} -\sum_{k=1}^{m}R^{N} \bigl(du(X), du(e_{k}) \bigr)du(e_{k})+du \bigl( \operatorname{Ric}^{M}(X) \bigr)=\triangle du(X)+\widetilde{\nabla }^{2}du(X), \end{aligned}$$

where *X* is any smooth vector field on $M^{m}$. With respect to the variational vector field $du(V_{A})$ along *u*, it follows from (13) and (14) that

15 $$\begin{aligned}& \sum_{A}I \bigl(du(V_{A}),du(V_{A}) \bigr) \\& \quad = \int_{M}F'' \biggl(\frac{\Vert u^{*}h \Vert ^{2}}{4} \biggr)\sum_{A} \bigl\langle \widetilde{\nabla } du(V_{A}),\sigma_{u}\bigr\rangle ^{2} \,dv_{g} \\& \quad \quad {}+ \int_{M}F' \biggl(\frac{\Vert u^{*}h \Vert ^{2}}{4} \biggr)\sum _{i,j,A}h \bigl( \widetilde{\nabla }_{e_{i}} du(V_{A}),\widetilde{\nabla }_{e_{j}}du(V _{A}) \bigr)h \bigl(du(e_{i}),du(e_{j}) \bigr)\,dv_{g} \\& \quad \quad {}+ \int_{M}F' \biggl(\frac{\Vert u^{*}h \Vert ^{2}}{4} \biggr)\sum _{i,j,A}h \bigl( \widetilde{\nabla }_{e_{i}} du(V_{A}),du(e_{j}) \bigr)h \bigl(\widetilde{\nabla } _{e_{i}}du(V_{A}),du(e_{j}) \bigr) \,dv_{g} \\& \quad \quad {}+ \int_{M}F' \biggl(\frac{\Vert u^{*}h \Vert ^{2}}{4} \biggr)\sum _{i,j,A}h \bigl( \widetilde{\nabla }_{e_{i}} du(V_{A}),du(e_{j}) \bigr)h \bigl(du(e_{i}), \widetilde{\nabla }_{e_{j}}du(V_{A}) \bigr)\,dv_{g} \\& \quad \quad {}- \int_{M}F' \biggl(\frac{\Vert u^{*}h \Vert ^{2}}{4} \biggr)\sum _{A}h \bigl(du \bigl(\operatorname{Ric}^{M^{m}}(V_{A}) \bigr), \sigma_{u}(V_{A}) \bigr)\,dv_{g} \\& \quad \quad {}+ \int_{M}F' \biggl(\frac{\Vert u^{*}h \Vert ^{2}}{4} \biggr)\sum _{A}h \bigl( \bigl(\widetilde{\nabla }^{2} du \bigr) (V_{A}),\sigma_{u}(V_{A}) \bigr) \,dv_{g}. \end{aligned}$$

For any fixed point $P\in M$, choose $\{e_{i}\}$ such that $\nabla _{e_{i}}e_{j}|_{P}=0$. We have

16 $$\begin{aligned} \widetilde{\nabla }^{2}du(V_{A})= \widetilde{\nabla }_{e_{i}} \widetilde{\nabla }_{e_{i}} \bigl(du(V_{A}) \bigr)-2\widetilde{\nabla }_{e_{i}} \bigl(du( \nabla_{e_{i}}V_{A}) \bigr) +du(\nabla_{e_{i}} \nabla_{e_{i}}V_{A}) \end{aligned}$$

and

17 $$\begin{aligned} & \int_{M}F' \biggl(\frac{\Vert u^{*}h \Vert ^{2}}{4} \biggr)\sum _{A,i}h \bigl(\widetilde{\nabla } _{e_{i}} \widetilde{\nabla }_{e_{i}}du(V_{A}),\sigma_{u}(V_{A}) \bigr)\,dv_{g} \\ &\quad =- \int_{M}\sum_{A,i}h \biggl( \widetilde{\nabla }_{e_{i}}du(V_{A}), \widetilde{\nabla }_{e_{i}} \biggl[F' \biggl(\frac{\Vert u^{*}h \Vert ^{2}}{4} \biggr) \sigma_{u}(V _{A}) \biggr] \biggr)\,dv_{g} \\ &\quad =- \int_{M}\sum_{A,i}h \biggl( \widetilde{\nabla }_{e_{i}}du(V_{A}), \widetilde{\nabla }_{e_{i}} \biggl[F' \biggl(\frac{\Vert u^{*}h \Vert ^{2}}{4} \biggr) \biggr] \sigma_{u}(V _{A}) \biggr)\,dv_{g} \\ &\quad \quad {}- \int_{M}\sum_{A,i}h \biggl( \widetilde{\nabla }_{e_{i}}du(V_{A}),F' \biggl( \frac{\Vert u^{*}h \Vert ^{2}}{4} \biggr)\widetilde{\nabla }_{e_{i}}\sigma_{u}(V_{A}) \biggr)\,dv_{g} \\ &\quad =- \int_{M}\sum_{A,i}h \biggl( \widetilde{\nabla }_{e_{i}}du(V_{A}), \widetilde{\nabla }_{e_{i}} \biggl[F' \biggl(\frac{\Vert u^{*}h \Vert ^{2}}{4} \biggr) \biggr] \sigma_{u}(V _{A}) \biggr)\,dv_{g} \\ &\quad \quad {}- \int_{M}F' \biggl(\frac{\Vert u^{*}h \Vert ^{2}}{4} \biggr)\sum _{A,i,j}h \bigl( \widetilde{\nabla }_{e_{i}} du(V_{A}),\widetilde{\nabla }_{e_{i}}du(e _{j}) \bigr)h \bigl(du(V_{A}),du(e_{j}) \bigr)\,dv_{g} \\ &\quad \quad {}- \int_{M}F' \biggl(\frac{\Vert u^{*}h \Vert ^{2}}{4} \biggr)\sum _{A,i,j}h \bigl( \widetilde{\nabla }_{e_{i}} du(V_{A}),du(e_{j}) \bigr)h \bigl(\widetilde{\nabla } _{e_{i}}du(V_{A}),du(e_{j}) \bigr) \,dv_{g} \\ &\quad \quad {}- \int_{M}F' \biggl(\frac{\Vert u^{*}h \Vert ^{2}}{4} \biggr)\sum _{A,i,j}h \bigl( \widetilde{\nabla }_{e_{i}} du(V_{A}),du(e_{j}) \bigr)h \bigl(du(V_{A}), \widetilde{\nabla }_{e_{i}}du(e_{j}) \bigr) \,dv_{g}. \end{aligned}$$

Substituting (16) and (17) into (15), we have

18 $$\begin{aligned} &\sum_{A}I \bigl(du(V_{A}),du(V_{A}) \bigr) \\ &\quad = \int_{M} \biggl\{ F'' \biggl( \frac{\Vert u^{*}h \Vert ^{2}}{4} \biggr)\sum_{A} \bigl\langle \widetilde{\nabla }du(V_{A}),\sigma _{u} \bigr\rangle ^{2} \\ &\quad \quad {}-h \biggl(\widetilde{\nabla }_{e_{i}} du(V_{A}),\widetilde{ \nabla }_{e_{i}} \biggl[F' \biggl(\frac{\Vert u^{*}h \Vert ^{2}}{4} \biggr) \biggr]\sigma_{u}(V_{A}) \biggr) \biggr\} \,dv_{g} \\ &\quad \quad {}+ \int_{M}F' \biggl(\frac{\Vert u^{*}h \Vert ^{2}}{4} \biggr)h \bigl(-2\widetilde{\nabla }_{e_{i}} \bigl(du( \nabla_{e_{i}}V_{A}) \bigr) \\ &\quad \quad {}+du(\nabla_{e_{i}}\nabla_{e_{i}}V_{A})-du \bigl(\operatorname{Ric}^{M^{m}}(V_{A}) \bigr),\sigma _{u}(V_{A}) \bigr)\,dv_{g} \\ &\quad \quad {}+ \int_{M}F' \biggl(\frac{\Vert u^{*}h \Vert ^{2}}{4} \biggr)\sum _{i,j,A}h \bigl( \widetilde{\nabla }_{e_{i}} du(V_{A}),\widetilde{\nabla }_{e_{j}}du(V _{A}) \bigr)h \bigl(du(e_{i}),du(e_{j}) \bigr)\,dv_{g} \\ &\quad \quad {}+ \int_{M}F' \biggl(\frac{\Vert u^{*}h \Vert ^{2}}{4} \biggr)\sum _{i,j,A}h \bigl( \widetilde{\nabla }_{e_{i}} du(V_{A}),du(e_{j}) \bigr)h \bigl(du(e_{i}), \widetilde{\nabla }_{e_{j}}du(V_{A}) \bigr)\,dv_{g} \\ &\quad \quad {}- \int_{M}F' \biggl(\frac{\Vert u^{*}h \Vert ^{2}}{4} \biggr)\sum _{A,i,j}h \bigl( \widetilde{\nabla }_{e_{i}} du(V_{A}),\widetilde{\nabla }_{e_{i}}du(e _{j}) \bigr)h \bigl(du(V_{A}),du(e_{j}) \bigr)\,dv_{g} \\ &\quad \quad {}- \int_{M}F' \biggl(\frac{\Vert u^{*}h \Vert ^{2}}{4} \biggr)\sum _{A,i,j}h \bigl( \widetilde{\nabla }_{e_{i}} du(V_{A}),du(e_{j}) \bigr)h \bigl(du(V_{A}), \widetilde{\nabla }_{e_{i}}du(e_{j}) \bigr) \,dv_{g}. \end{aligned}$$

In the following, we shall estimate each term in (18). Because trace is independent of the choice of orthonormal basis, we can take pointwisely $\{e_{i},e_{m+1}\}$ such that $B_{ij}=\lambda_{i}\delta _{ij}$.

A straightforward computation shows

19 $$\begin{aligned} &\sum_{A}h \biggl(\widetilde{ \nabla }_{e_{i}}du(V_{A}),\widetilde{\nabla } _{e_{i}} \biggl[F' \biggl(\frac{\Vert u^{*}h \Vert ^{2}}{4} \biggr) \biggr] \sigma_{u}(V_{A}) \biggr) \\ &\quad =\sum_{A}F'' \biggl( \frac{\Vert u^{*}h \Vert ^{2}}{4} \biggr)\widetilde{\nabla }_{e_{i}} \biggl( \frac{\Vert u^{*}h \Vert ^{2}}{4} \biggr)h \bigl(v_{A}^{m+1}B_{ik} du(e_{k}) +v_{A}^{k} \widetilde{\nabla }_{e_{i}} du(e_{k}),v_{A}^{l} \sigma_{u}(e_{l}) \bigr) \\ &\quad =F'' \biggl(\frac{\Vert u^{*}h \Vert ^{2}}{4} \biggr) \widetilde{\nabla }_{e_{i}} \biggl(\frac{\Vert u^{*}h \Vert ^{2}}{4} \biggr)h \bigl( \widetilde{\nabla }_{e_{i}}du(e_{k}),\sigma_{u}(e _{k}) \bigr) \\ &\quad =F'' \biggl(\frac{\Vert u^{*}h \Vert ^{2}}{4} \biggr) \langle \widetilde{ \nabla }_{e_{i}}du,\sigma _{u} \rangle^{2} \end{aligned}$$

and

20 $$\begin{aligned} &\sum_{A}F'' \biggl( \frac{\Vert u^{*}h \Vert ^{2}}{4} \biggr) \bigl\langle \widetilde{\nabla } du(V_{A}),\sigma_{u} \bigr\rangle ^{2} \\ &\quad =\sum_{A}F''\biggl( \frac{\Vert u^{*}h \Vert ^{2}}{4} \biggr) \bigl\langle v_{A}^{m+1}B_{ik} du(e_{k})+v _{A}^{k}\widetilde{\nabla}_{e_{i}}du(e_{k}),\sigma_{u}(e_{i} )\bigr\rangle ^{2} \\ &\quad =\sum_{A}F'' \biggl( \frac{\Vert u^{*}h \Vert ^{2}}{4} \biggr) \bigl\{ B_{ik}B_{jl}h \bigl(du(e_{k}), \sigma_{u}(e_{i}) \bigr)h \bigl(du(e_{l}),\sigma_{u}(e_{j}) \bigr) \\ &\quad \quad {}+h \bigl(\widetilde{\nabla }_{e_{i}}du(e_{k}), \sigma_{u}(e_{i}) \bigr)h \bigl( \widetilde{\nabla }_{e_{j}}du(e_{k}),\sigma_{u}(e_{j}) \bigr) \bigr\} \\ &\quad =\sum_{A}F'' \biggl( \frac{\Vert u^{*}h \Vert ^{2}}{4} \biggr) \bigl\{ \lambda_{i} \lambda_{j} h \bigl(du(e _{i}),\sigma_{u}(e_{i}) \bigr)h \bigl(du(e_{j}),\sigma_{u}(e_{j}) \bigr) +\langle \widetilde{\nabla }_{e_{i}}du,\sigma_{u} \rangle^{2} \bigr\} . \end{aligned}$$

Then it follows from (19) and (20) that

21 $$\begin{aligned} & \int_{M} \biggl\{ F'' \biggl( \frac{\Vert u^{*}h \Vert ^{2}}{4} \biggr)\sum_{A} \bigl\langle \widetilde{\nabla }du(V _{A}),\sigma_{u} \bigr\rangle ^{2} \\ &\quad \quad {}-h \biggl(\widetilde{\nabla }_{e_{i}} du(V_{A}),\widetilde{\nabla }_{e_{i}} \biggl[F' \biggl(\frac{\Vert u^{*}h \Vert ^{2}}{4} \biggr) \biggr]\sigma_{u}(V_{A}) \biggr) \biggr\} \,dv_{g} \\ &= \int_{M}F'' \biggl(\frac{\Vert u^{*}h \Vert ^{2}}{4} \biggr)\lambda_{i}\lambda_{j} h \bigl(du(e _{i}), \sigma_{u}(e_{i}) \bigr)h \bigl(du(e_{j}), \sigma_{u}(e_{j}) \bigr) \,dv_{g}. \end{aligned}$$

From the Gauss equation it follows that

22 $$\begin{aligned} \operatorname{Ric}^{M}(V_{A})=v_{A}^{i}(B_{kk}B_{ij}-B_{ik}B_{jk})e_{j}. \end{aligned}$$

Using (10), (11),(12) and (22), we have

23 $$\begin{aligned} & \int_{M}F' \biggl(\frac{\Vert u^{*}h \Vert ^{2}}{4} \biggr)h \bigl(-2\widetilde{\nabla }_{e_{i}} \bigl(du( \nabla_{e_{i}}V_{A}) \bigr) \\ &\quad \quad {}+du(\nabla_{e_{i}}\nabla_{e_{i}}V_{A})-du \bigl( \operatorname{Ric}^{M^{m}}(V_{A}) \bigr),\sigma _{u}(V_{A}) \bigr)\,dv_{g} \\ &\quad = \int_{M}F' \biggl(\frac{\Vert u^{*}h \Vert ^{2}}{4} \biggr) \bigl\{ \bigl[h \bigl(2v_{A}^{k}B_{ik}B_{ij} du(e _{j})-v_{A}^{m+1}\nabla_{e_{i}}(B_{ij}) du(e_{j}) \\ &\quad \quad {}-v_{A}^{m+1}B_{ij}\widetilde{\nabla }_{e_{i}}du(e_{j}),v_{A}^{l} \sigma_{u}(e_{l}) \bigr) \bigr] \\ &\quad \quad {}+h \bigl(-v_{A}^{k}B_{ik}B_{ij} du(e_{j})+v_{A}^{m+1}(\nabla_{e_{i}}B_{ij}) du(e _{j}),v_{A}^{l}\sigma_{u}(e_{l}) \bigr) \\ &\quad \quad {}+h \bigl(v_{A}^{k}B_{ik}B_{ij} du(e_{j})-v^{i}_{A}B_{kk}B_{ij} du(e_{j}),v _{A}^{l}\sigma_{u}(e_{l}) \bigr) \bigr\} \,dv_{g} \\ &\quad =\int_{M}F' \biggl(\frac{\Vert u^{*}h \Vert ^{2}}{4} \biggr) \bigl\{ h\bigl(2v_{A}^{k}B_{ik}B_{ij}du(e_{j})-v^{i}_{A}B_{kk}B_{ij}du(e_{j}),v_{A}^{l}\sigma_{u}(e_{l})\bigr)\bigr\} \,dv _{g} \\ &\quad =\int_{M}F' \biggl(\frac{\Vert u^{*}h \Vert ^{2}}{4} \biggr)\sum_{i} \biggl\{ \biggl[2\lambda_{i}-\biggl( \sum_{k}\lambda_{k} \biggr) \biggr] \lambda_{i}h \bigl(du(e_{i}),\sigma_{u}(e_{i}) \bigr) \biggr\} \,dv_{g}. \end{aligned}$$

A straightforward computation shows

24 $$\begin{aligned} & \int_{M}F' \biggl(\frac{\Vert u^{*}h \Vert ^{2}}{4} \biggr)\sum _{i,j,A}h \bigl( \widetilde{\nabla }_{e_{i}} du(V_{A}),\widetilde{\nabla }_{e_{j}}du(V _{A}) \bigr)h \bigl(du(e_{i}),du(e_{j}) \bigr)\,dv_{g} \\ &\quad = \int_{M}F' \biggl(\frac{\Vert u^{*}h \Vert ^{2}}{4} \biggr)h \bigl(v_{A}^{m+1}B_{ik}du(e_{k})+v _{A}^{k}\widetilde{\nabla }_{e_{i}} du(e_{k}), \\ &\quad \quad v_{A}^{m+1}B_{jl}du(e_{l})+v_{A}^{l} \widetilde{\nabla }_{e_{j}}du(e _{l}) \bigr)h \bigl(du(e_{i}),du(e_{j}) \bigr)\,dv_{g} \\ &\quad = \int_{M}F' \biggl(\frac{\Vert u^{*}h \Vert ^{2}}{4} \biggr) \bigl\{ B_{ik}B_{jl}h \bigl(du(e_{k}),du(e _{l}) \bigr)h \bigl(du(e_{i}),du(e_{j}) \bigr) \\ &\quad \quad {}+h \bigl(\widetilde{\nabla }_{e_{i}}du(e_{k}), \widetilde{ \nabla }_{e_{j}}du(e _{k}) \bigr)h \bigl(du(e_{i}),du(e_{j}) \bigr) \bigr\} \,dv_{g} \\ &\quad = \int_{M}F' \biggl(\frac{\Vert u^{*}h \Vert ^{2}}{4} \biggr) \bigl\{ \lambda_{i}\lambda_{j}h \bigl(du(e _{i}),du(e_{j}) \bigr)h \bigl(du(e_{i}),du(e_{j}) \bigr) \\ &\quad \quad {}+h \bigl(\widetilde{\nabla }_{e_{i}}du(e_{k}), \widetilde{ \nabla }_{e_{j}}du(e _{k}) \bigr)h \bigl(du(e_{i}),du(e_{j}) \bigr) \bigr\} \,dv_{g} \end{aligned}$$

and

25 $$\begin{aligned} & \int_{M}F' \biggl(\frac{\Vert u^{*}h \Vert ^{2}}{4} \biggr)\sum _{i,j,A}h \bigl( \widetilde{\nabla }_{e_{i}} du(V_{A}),du(e_{j}) \bigr)h \bigl(du(e_{i}), \widetilde{\nabla }_{e_{j}}du(V_{A}) \bigr)\,dv_{g} \\ &\quad = \int_{M}F' \biggl(\frac{\Vert u^{*}h \Vert ^{2}}{4} \biggr) \bigl\{ h \bigl(v_{A}^{m+1}B_{ik}du(e_{k})+v _{A}^{k}\widetilde{\nabla }_{e_{i}} du(e_{k}),du(e_{j}) \bigr) \\ &\quad \quad {}\times h \bigl(du(e_{i}),v_{A}^{m+1}B_{jk} du(e_{k})+v_{A}^{k}\widetilde{\nabla } _{e_{j}}du(e_{k}) \bigr) \bigr\} \,dv_{g} \\ &\quad = \int_{M}F' \biggl(\frac{\Vert u^{*}h \Vert ^{2}}{4} \biggr) \bigl\{ \lambda_{i}\lambda_{j}h \bigl(du(e _{i}),du(e_{j}) \bigr)h \bigl(du(e_{i}),du(e_{j}) \bigr) \\ &\quad \quad {}+h \bigl(\widetilde{\nabla }_{e_{i}}du(e_{k}),du(e_{j}) \bigr)h \bigl( \widetilde{\nabla }_{e_{j}}du(e_{k}),du(e_{i}) \bigr) \bigr\} \,dv_{g} \end{aligned}$$

and

26 $$\begin{aligned} & \int_{M}F' \biggl(\frac{\Vert u^{*}h \Vert ^{2}}{4} \biggr)\sum _{A,i,j}h \bigl( \widetilde{\nabla }_{e_{i}} du(V_{A}),\widetilde{\nabla }_{e_{i}}du(e _{j}) \bigr)h \bigl(du(V_{A}),du(e_{j}) \bigr)\,dv_{g} \\ &\quad = \int_{M}F' \biggl(\frac{\Vert u^{*}h \Vert ^{2}}{4} \biggr) \bigl\{ h \bigl(v_{A}^{m+1}B_{ik}du(e_{k})+v _{A}^{k}\widetilde{\nabla }_{e_{i}} du(e_{k}), \widetilde{\nabla }_{e _{i}}du(e_{j}) \bigr) \\ &\quad \quad {}\times h \bigl(v_{A}^{l} du(e_{l}),du(e_{j}) \bigr) \bigr\} \,dv_{g} \\ &\quad = \int_{M}F' \biggl(\frac{\Vert u^{*}h \Vert ^{2}}{4} \biggr)h \bigl( \widetilde{\nabla }_{e_{i}}du(e _{k}), \widetilde{ \nabla }_{e_{i}}du(e_{j}) \bigr)h \bigl(du(e_{k}),du(e_{j}) \bigr)\,dv _{g} \end{aligned}$$

and

27 $$\begin{aligned} & \int_{M}F' \biggl(\frac{\Vert u^{*}h \Vert ^{2}}{4} \biggr)\sum _{A,i,j}h \bigl( \widetilde{\nabla }_{e_{i}} du(V_{A}),du(e_{j}) \bigr)h \bigl(du(V_{A}), \widetilde{\nabla }_{e_{i}}du(e_{j}) \bigr)\,dv_{g} \\ &\quad = \int_{M}F' \biggl(\frac{\Vert u^{*}h \Vert ^{2}}{4} \biggr) \bigl\{ h \bigl(v_{A}^{m+1}B_{ik}du(e_{k})+v _{A}^{k}\widetilde{\nabla }_{e_{i}} du(e_{k}),du(e_{j}) \bigr) \\ &\quad \quad {}\times h \bigl(v_{A}^{l} du(e_{l}), \widetilde{\nabla }_{e_{i}}du(e_{j}) \bigr) \bigr\} \,dv _{g} \\ &\quad = \int_{M}F' \biggl(\frac{\Vert u^{*}h \Vert ^{2}}{4} \biggr)h \bigl( \widetilde{\nabla }_{e_{i}}du(e _{k}),du(e_{j}) \bigr)h \bigl(du(e_{k}),\widetilde{\nabla }_{e_{i}} du(e_{j}) \bigr)\,dv _{g}. \end{aligned}$$

From (18), (21), (23), (24), (25), (26), (27) and $\widetilde{\nabla }_{e_{i}}du(e_{j})=\widetilde{\nabla }_{e_{j}}du(e _{i})$, we obtain

28 $$\begin{aligned} &\sum_{A}I \bigl(du(V_{A}),du(V_{A}) \bigr) \\ &\quad = \int_{M}F'' \biggl(\frac{\Vert u^{*}h \Vert ^{2}}{4} \biggr)\lambda_{i}\lambda_{j} h \bigl(du(e _{i}), \sigma_{u}(e_{i}) \bigr)h \bigl(du(e_{j}), \sigma_{u}(e_{j}) \bigr) \,dv_{g} \\ &\quad \quad {}+ \int_{M}F' \biggl(\frac{\Vert u^{*}h \Vert ^{2}}{4} \biggr)\sum _{i} \biggl\{ \biggl[2\lambda_{i}- \biggl( \sum_{k}\lambda_{k} \biggr) \biggr] \lambda_{i}h \bigl(du(e_{i}),\sigma_{u}(e_{i}) \bigr) \biggr\} \,dv_{g} \\ &\quad \quad {}+2 \int_{M}F' \biggl(\frac{\Vert u^{*}h \Vert ^{2}}{4} \biggr) \lambda_{i}\lambda_{j}h \bigl(du(e _{i}),du(e_{j}) \bigr)h \bigl(du(e_{i}),du(e_{j}) \bigr)\,dv_{g} \\ &\quad \leq \int_{M}F'' \biggl(\frac{\Vert u^{*}h \Vert ^{2}}{4} \biggr)\lambda_{i}\lambda_{j} h \bigl(du(e _{i}), \sigma_{u}(e_{i}) \bigr)h \bigl(du(e_{j}), \sigma_{u}(e_{j}) \bigr) \,dv_{g} \\ &\quad \quad {}+ \int_{M}F' \biggl(\frac{\Vert u^{*}h \Vert ^{2}}{4} \biggr)\sum _{i} \biggl\{ \biggl[2\lambda_{i}- \biggl( \sum_{k}\lambda_{k} \biggr) \biggr] \lambda_{i}h \bigl(du(e_{i}),\sigma_{u}(e_{i}) \bigr) \biggr\} \,dv_{g} \\ &\quad \quad {}+2 \int_{M}F' \biggl(\frac{\Vert u^{*}h \Vert ^{2}}{4} \biggr) \lambda_{i}\lambda_{m}h \bigl(du(e _{i}), \sigma_{u}(e_{i}) \bigr)\,dv_{g} \\ &\quad = \int_{M}F'' \biggl(\frac{\Vert u^{*}h \Vert ^{2}}{4} \biggr)\lambda_{i}\lambda_{j} h \bigl(du(e _{i}), \sigma_{u}(e_{i}) \bigr)h \bigl(du(e_{j}), \sigma_{u}(e_{j}) \bigr) \,dv_{g} \\ &\quad \quad {}+ \int_{M}F' \biggl(\frac{\Vert u^{*}h \Vert ^{2}}{4} \biggr)\sum _{i} \biggl\{ \biggl[2\lambda_{i}+2 \lambda_{m}- \biggl(\sum_{k} \lambda_{k} \biggr) \biggr]\lambda_{i}h \bigl(du(e_{i}), \sigma_{u}(e _{i}) \bigr) \biggr\} \,dv_{g}. \end{aligned}$$

If $F''(t)=F'(t)$, then (28) leads to the following inequality:

29 $$\begin{aligned} &\sum_{A}I \bigl(du(V_{A}),du(V_{A}) \bigr) \\ &\quad \leq \int_{M}F' \biggl(\frac{\Vert u^{*}h \Vert ^{2}}{4} \biggr) \lambda_{m}^{2} \bigl\Vert u^{*}h \bigr\Vert ^{4}\,dv _{g} \\ &\quad \quad {}+ \int_{M}F' \biggl(\frac{\Vert u^{*}h \Vert ^{2}}{4} \biggr)\max _{1\leq i\leq m} \biggl\{ \biggl[2\lambda _{i}+2 \lambda_{m}- \biggl(\sum_{k} \lambda_{k} \biggr) \biggr]\lambda_{i} \biggr\} \bigl\Vert u^{*}h \bigr\Vert ^{2}\,dv _{g} \\ &\quad = \int_{M}F' \biggl(\frac{\Vert u^{*}h \Vert ^{2}}{4} \biggr) \bigl\Vert u^{*}h \bigr\Vert ^{2} \biggl\{ \lambda_{m} ^{2} \bigl\Vert u^{*}h \bigr\Vert ^{2} \\ &\quad \quad {}+\max_{1\leq i\leq m} \biggl\{ \biggl[2 \lambda_{i}+2 \lambda_{m}- \biggl( \sum _{k} \lambda_{k} \biggr) \biggr] \lambda_{i} \biggr\} \biggr\} \,dv_{g}. \end{aligned}$$

If there exists a constant $c_{F}$ such that $\frac{F'(t)}{t^{c_{F}}}$ is nonincreasing, it follows that $F''(t)t\leq c_{F}F'(t)$ on $t\in (0,\infty )$, thus (28) implies

30 $$\begin{aligned} &\sum_{A}I \bigl(du(V_{A}),du(V_{A}) \bigr) \\ &\quad \leq \int_{M}4c_{F}F' \biggl( \frac{\Vert u^{*}h \Vert ^{2}}{4} \biggr)\lambda_{m}^{2} \bigl\Vert u^{*}h \bigr\Vert ^{2}\,dv_{g} \\ &\quad \quad {}+ \int_{M}F' \biggl(\frac{\Vert u^{*}h \Vert ^{2}}{4} \biggr)\max _{1\leq i\leq m} \biggl\{ \biggl[2\lambda _{i}+2 \lambda_{m}- \biggl(\sum_{k} \lambda_{k} \biggr) \biggr]\lambda_{i} \biggr\} \bigl\Vert u^{*}h \bigr\Vert ^{2}\,dv _{g} \\ &\quad = \int_{M}F' \biggl(\frac{\Vert u^{*}h \Vert ^{2}}{4} \biggr) \bigl\Vert u^{*}h \bigr\Vert ^{2} \biggl\{ 4c_{F} \lambda _{m}^{2} \\ &\quad \quad {}+\max_{1\leq i\leq m} \biggl\{ \biggl[2 \lambda_{i}+2\lambda_{m}- \biggl(\sum _{k} \lambda_{k} \biggr) \biggr] \lambda_{i} \biggr\} \biggr\} \,dv_{g}. \end{aligned}$$

If *u* is nonconstant and (6) or (7) holds, we have

31 $$\begin{aligned} \sum_{A}I \bigl(du(V_{A}),du(V_{A}) \bigr)< 0 \end{aligned}$$

and *u* is unstable. □

### Corollary 3.2

*Let* $u:S^{m}\rightarrow N$ *be a nonconstant* *F*-*stationary map and* $m>4$. *If* $c_{F}<\frac{m}{4}-1$ *or* $\Vert u^{*}h \Vert ^{2}< m-4$, *then* *u* *is unstable*.

## *F*-stationary maps into compact convex hypersurfaces

In this section, we obtain the following result.

### Theorem 4.1

*With the same assumption on* $M^{m}$ *as in Theorem* 3.1, *every nonconstant* *F*-*stationary map from any compact Riemannian manifold* *N* *to* $M^{m}$ *is unstable if* (6) *or* (7) *holds*.

### Proof

In order to prove the instability of $u:N^{n}\rightarrow M^{m}$, we need to consider some special variational vector fields along *u*. To do this, we choose an orthonormal field $\{\epsilon_{\alpha },\epsilon _{m+1}\}$, $\alpha =1,\ldots ,m$, of $R^{m+1}$ such that $\{ \epsilon_{\alpha }\}$ are tangent to $M^{m}\subset R^{m+1}$, $\epsilon_{m+1}$ is normal to $M^{m}$, $^{M^{m}} \nabla_{\epsilon_{\alpha }}\epsilon_{\beta }|_{P}=0$ and $B_{\alpha \beta }=\lambda_{\alpha }\delta_{\alpha \beta }$, where $B_{\alpha \beta }$ denotes the components of the second fundamental form of $M^{m}$ in $R^{m+1}$. Meanwhile, take a fixed orthonormal basis $E_{A}$, $A=1,\ldots ,m+1$, of $R^{m+1}$ and set

32 $$\begin{aligned} V_{A}=\sum_{\alpha =1}^{m}v_{A}^{\alpha } \epsilon_{\alpha },\quad v_{A}^{\alpha }=\langle E_{A},\epsilon_{\alpha }\rangle, v_{A}^{m+1}= \langle E_{A},\epsilon_{m+1}\rangle, \end{aligned}$$

where $\langle \cdot, \cdot \rangle $ denotes the canonical Euclidean inner product. We shall consider the second variation

33 $$\begin{aligned} \sum_{A}I(V_{A},V_{A}) =& \int_{N}F'' \biggl(\frac{\Vert u^{*}h \Vert ^{2}}{4} \biggr) \langle \widetilde{\nabla }V_{A},\sigma_{u}\rangle \langle \widetilde{\nabla }V_{A},\sigma _{u}\rangle \,dv_{g} \\ & {}+ \int_{N}F' \biggl(\frac{\Vert u^{*}h \Vert ^{2}}{4} \biggr)\sum _{i,j=1}^{m}h( \widetilde{\nabla }_{e_{i}}V_{A},\widetilde{\nabla }_{e_{j}}V_{A})h \bigl(du(e _{i}),du(e_{j}) \bigr)\,dv_{g} \\ & {}+ \int_{N}F' \biggl(\frac{\Vert u^{*}h \Vert ^{2}}{4} \biggr)\sum _{i,j=1}^{m}h \bigl( \widetilde{\nabla }_{e_{i}}V_{A},du(e_{j}) \bigr)h \bigl(\widetilde{ \nabla }_{e _{i}}V_{A},du(e_{j}) \bigr) \,dv_{g} \\ & {}+ \int_{N}F' \biggl(\frac{\Vert u^{*}h \Vert ^{2}}{4} \biggr)\sum _{i,j=1}^{m}h \bigl( \widetilde{\nabla }_{e_{i}}V_{A},du(e_{j}) \bigr)h \bigl(du(e_{i}), \widetilde{\nabla }_{e_{j}}V_{A} \bigr)\,dv_{g} \\ &{}+ \int_{N}F' \biggl(\frac{\Vert u^{*}h \Vert ^{2}}{4} \biggr)\sum _{i}h \bigl(R^{M^{m}} \bigl(V_{A},du(e _{i}) \bigr)V_{A},\sigma_{u}(e_{i}) \bigr)\,dv_{g}, \end{aligned}$$

where $\{e_{1},\ldots ,e_{n}\}$ is the local orthonormal frame of $N^{n}$.

Firstly, we compute the first term of (33)

34 $$\begin{aligned} &\sum_{A} \int_{N}F'' \biggl(\frac{\Vert u^{*}h \Vert ^{2}}{4} \biggr) \langle \widetilde{\nabla }V _{A},\sigma_{u}\rangle \langle \widetilde{ \nabla }V_{A},\sigma_{u}\rangle\, dv_{g} \\ &\quad = \int_{N}F'' \biggl(\frac{\Vert u^{*}h \Vert ^{2}}{4} \biggr) \biggl[\sum_{i}h \bigl( \widetilde{\nabla }_{e_{i}}V_{A},\sigma_{u}(e_{i}) \bigr) \biggr]^{2}\,dv_{g} \\ &\quad = \int_{N}F'' \biggl(\frac{\Vert u^{*}h \Vert ^{2}}{4} \biggr) \biggl[\sum_{i}h \bigl({}^{M^{m}} \nabla_{du(e_{i})}V_{A},\sigma_{u}(e_{i}) \bigr) \biggr]^{2}\,dv_{g} \\ &\quad = \int_{N}F'' \biggl(\frac{\Vert u^{*}h \Vert ^{2}}{4} \biggr) \biggl[\sum_{i}u_{i}^{\alpha }h \bigl({}^{M ^{m}}\nabla_{\epsilon_{\alpha }}V_{A}, \sigma_{u}(e_{i}) \bigr) \biggr]^{2} \,dv_{g} \\ &\quad = \int_{N}F'' \biggl(\frac{\Vert u^{*}h \Vert ^{2}}{4} \biggr) \biggl[\sum_{i}v_{A}^{m+1}u_{i}^{ \alpha }B_{\alpha \beta } h \bigl(\epsilon_{\beta },\sigma_{u}(e_{i}) \bigr) \biggr]^{2}\,dv _{g} \\ &\quad = \int_{N}F'' \biggl(\frac{\Vert u^{*}h \Vert ^{2}}{4} \biggr) \biggl[\sum_{i}v_{A}^{m+1}u_{i}^{ \alpha } \lambda_{\alpha }h \bigl(\epsilon_{\alpha },\sigma_{u}(e_{i}) \bigr) \biggr]^{2}\,dv _{g} \\ &\quad = \int_{N}F'' \biggl(\frac{\Vert u^{*}h \Vert ^{2}}{4} \biggr) \lambda_{\alpha }\lambda_{ \beta }h \bigl(u_{i}^{\alpha } \epsilon_{\alpha },\sigma_{u}(e_{i}) \bigr)h \bigl(u_{j} ^{\beta }\epsilon_{\beta }, \sigma_{u}(e_{j}) \bigr)\,dv_{g}. \end{aligned}$$

The second term of (33)

35 $$\begin{aligned} &\sum_{A} \int_{N}F' \biggl(\frac{\Vert u^{*}h \Vert ^{2}}{4} \biggr)\sum _{i,j=1}^{m}h( \widetilde{\nabla }_{e_{i}}V_{A},\widetilde{\nabla }_{e_{j}}V_{A})h \bigl(du(e _{i}),du(e_{j}) \bigr)\,dv_{g} \\ &\quad =\int_{N}F' \biggl(\frac{\Vert u^{*}h \Vert {}^{2}}{4} \biggr)h \bigl({}^{M^{m}}\nabla_{du(e_{i})}V _{A},{}^{M^{m}} \nabla_{du(e_{j})}V_{A} \bigr)h \bigl(du(e_{i}),du(e_{j}) \bigr)\,dv_{g} \\ &\quad = \int_{N}F' \biggl(\frac{\Vert u^{*}h \Vert ^{2}}{4} \biggr)u_{i}^{\alpha }u_{j}^{\beta }h \bigl({}^{M ^{m}}\nabla_{\epsilon_{\alpha }}V_{A},{}^{M^{m}} \nabla_{\epsilon_{ \beta }}V_{A} \bigr)h \bigl(du(e_{i}),du(e_{j}) \bigr)\,dv_{g} \\ &\quad = \int_{N}F' \biggl(\frac{\Vert u^{*}h \Vert ^{2}}{4} \biggr)u_{i}^{\alpha }u_{j}^{\beta }B _{\alpha \gamma }B_{\beta \delta }h(\epsilon_{\gamma },\epsilon_{ \delta })h \bigl(du(e_{i}),du(e_{j}) \bigr)\,dv_{g} \\ &\quad = \int_{N}F' \biggl(\frac{\Vert u^{*}h \Vert ^{2}}{4} \biggr) \lambda_{\alpha }\lambda_{ \beta }h \bigl(u_{i}^{\alpha } \epsilon_{\alpha },u_{j}^{\beta }\epsilon_{ \beta } \bigr)h \bigl(du(e_{i}),du(e_{j}) \bigr)\,dv_{g} \\ &\quad = \int_{N}F' \biggl(\frac{\Vert u^{*}h \Vert ^{2}}{4} \biggr) \lambda_{\alpha }^{2} h \bigl(u_{i} ^{\alpha } \epsilon_{\alpha },du(e_{j}) \bigr)h \bigl(du(e_{i}),du(e_{j}) \bigr)\,dv_{g}. \end{aligned}$$

The third term of (33)

36 $$\begin{aligned} &\sum_{A} \int_{N}F' \biggl(\frac{\Vert u^{*}h \Vert ^{2}}{4} \biggr)\sum _{i,j=1}^{m}h \bigl( \widetilde{\nabla }_{e_{i}}V_{A},du(e_{j}) \bigr)h \bigl(\widetilde{ \nabla }_{e _{i}}V_{A},du(e_{j}) \bigr) \,dv_{g} \\ &\quad = \int_{N}F' \biggl(\frac{\Vert u^{*}h \Vert ^{2}}{4} \biggr) \lambda_{\alpha }\lambda_{ \beta }h \bigl(u_{i}^{\alpha } \epsilon_{\alpha },du(e_{j}) \bigr)h \bigl(u_{i}^{\beta } \epsilon_{\beta },du(e_{j}) \bigr)\,dv_{g}. \end{aligned}$$

The fourth term of (33)

37 $$\begin{aligned} &\sum_{A} \int_{N}F' \biggl(\frac{\Vert u^{*}h \Vert ^{2}}{4} \biggr)\sum _{i,j=1}^{m}h \bigl( \widetilde{\nabla }_{e_{i}}V_{A},du(e_{j}) \bigr)h \bigl(du(e_{i}), \widetilde{\nabla }_{e_{j}}V_{A} \bigr)\,dv_{g} \\ &\quad = \int_{N}F' \biggl(\frac{\Vert u^{*}h \Vert ^{2}}{4} \biggr) \lambda_{\alpha }\lambda_{ \beta }h \bigl(u_{i}^{\alpha } \epsilon_{\alpha },du(e_{j}) \bigr)h \bigl(du(e_{i}),u _{j}^{\beta }\epsilon_{\beta } \bigr)\,dv_{g}. \end{aligned}$$

The fifth term of (33)

38 $$\begin{aligned} &\sum_{A} \int_{N}F' \biggl(\frac{\Vert u^{*}h \Vert ^{2}}{4} \biggr)\sum _{i}h \bigl(R^{M^{m}} \bigl(V _{A},du(e_{i}) \bigr)V_{A},\sigma_{u}(e_{i}) \bigr)\,dv_{g} \\ &\quad = \int_{N}F' \biggl(\frac{\Vert u^{*}h \Vert ^{2}}{4} \biggr)v_{A}^{\alpha }v_{A}^{\beta }h \bigl(R ^{M^{m}} \bigl(\epsilon_{\alpha },du(e_{i}) \bigr) \epsilon_{\beta },\sigma_{u}(e _{i}) \bigr) \,dv_{g} \\ &\quad = \int_{N}F' \biggl(\frac{\Vert u^{*}h \Vert ^{2}}{4} \biggr)u_{i}^{\gamma }u_{j}^{\delta }h \bigl(R ^{M^{m}}(\epsilon_{\alpha },\epsilon_{\gamma }) \epsilon_{\alpha }, \epsilon_{\delta } \bigr)h \bigl(du(e_{i}),du(e_{j}) \bigr)\,dv_{g} \\ &\quad = \int_{N}F' \biggl(\frac{\Vert u^{*}h \Vert ^{2}}{4} \biggr)u_{i}^{\gamma }u_{j}^{\delta }[B _{\alpha \delta }B_{\gamma \alpha }-B_{\alpha \alpha }B_{\gamma \delta }]h \bigl(du(e_{i}),du(e_{j}) \bigr)\,dv_{g} \\ &\quad = \int_{N}F' \biggl(\frac{\Vert u^{*}h \Vert ^{2}}{4} \biggr)u_{i}^{\alpha }u_{j}^{\alpha } \biggl[ \lambda_{\alpha }^{2}- \biggl(\sum_{\beta } \lambda_{\beta } \biggr) \lambda_{\alpha } \biggr]h \bigl(du(e_{i}),du(e_{j}) \bigr)\,dv_{g} \\ &\quad = \int_{N}F' \biggl(\frac{\Vert u^{*}h \Vert ^{2}}{4} \biggr) \biggl[ \lambda_{\alpha }^{2}- \biggl( \sum _{\beta } \lambda_{\beta } \biggr) \lambda_{\alpha } \biggr]h \bigl(u_{i}^{\alpha } \epsilon_{\alpha },u_{j}^{\gamma } \epsilon_{\gamma } \bigr)h \bigl(du(e_{i}),du(e _{j}) \bigr)\,dv_{g} \\ &\quad = \int_{N}F' \biggl(\frac{\Vert u^{*}h \Vert ^{2}}{4} \biggr) \biggl[ \lambda_{\alpha }^{2}- \biggl( \sum _{\beta } \lambda_{\beta } \biggr) \lambda_{\alpha } \biggr]h \bigl(u_{i}^{\alpha } \epsilon_{\alpha },du(e_{j}) \bigr)h \bigl(du(e_{i}),du(e_{j}) \bigr) \,dv_{g}. \end{aligned}$$

From (33)-(38), we have

39 $$\begin{aligned} &\sum_{A}I(V_{A},V_{A}) \\ &\quad = \int_{N}F'' \biggl(\frac{\Vert u^{*}h \Vert ^{2}}{4} \biggr) \lambda_{\alpha }\lambda_{\beta }h \bigl(u_{i}^{\alpha } \epsilon_{\alpha }, \sigma_{u}(e_{i}) \bigr)h \bigl(u_{j}^{\alpha }\epsilon_{\alpha },\sigma_{u}(e _{j}) \bigr)\,dv_{g} \\ &\quad \quad {}+ \int_{N}F' \biggl(\frac{\Vert u^{*}h \Vert ^{2}}{4} \biggr) \biggl[2 \lambda_{\alpha }^{2}- \biggl( \sum _{\beta } \lambda_{\beta } \biggr) \lambda_{\alpha } \biggr]h \bigl(u_{i}^{\alpha } \epsilon_{\alpha },du(e_{j}) \bigr)h \bigl(du(e_{i}),du(e_{j}) \bigr)\,dv_{g} \\ &\quad \quad {}+ \int_{N}F' \biggl(\frac{\Vert u^{*}h \Vert ^{2}}{4} \biggr) \lambda_{\alpha }\lambda_{ \beta }h \bigl(u_{i}^{\alpha } \epsilon_{\alpha },du(e_{j}) \bigr)h \bigl(u_{i}^{\beta } \epsilon_{\beta },du(e_{j}) \bigr)\,dv_{g} \\ &\quad \quad {}+ \int_{N}F' \biggl(\frac{\Vert u^{*}h \Vert ^{2}}{4} \biggr) \lambda_{\alpha }\lambda_{ \beta }h \bigl(u_{i}^{\alpha } \epsilon_{\alpha },du(e_{j}) \bigr)h \bigl(du(e_{i}),u _{j}^{\beta }\epsilon_{\beta } \bigr)\,dv_{g} \\ &\quad \leq \int_{N}F'' \biggl(\frac{\Vert u^{*}h \Vert ^{2}}{4} \biggr) \lambda_{\alpha } \lambda_{\beta }h \bigl(u_{i}^{\alpha } \epsilon_{\alpha },\sigma_{u}(e_{i}) \bigr)h \bigl(u _{j}^{\alpha }\epsilon_{\alpha },\sigma_{u}(e_{j}) \bigr)\,dv_{g} \\ &\quad \quad {}+ \int_{N}F' \biggl(\frac{\Vert u^{*}h \Vert ^{2}}{4} \biggr) \biggl[2 \lambda_{\alpha }^{2}- \biggl( \sum _{\beta } \lambda_{\beta } \biggr) \lambda_{\alpha } \biggr]h \bigl(u_{i}^{\alpha } \epsilon_{\alpha },du(e_{j}) \bigr)h \bigl(du(e_{i}),du(e_{j}) \bigr)\,dv_{g} \\ &\quad \quad {}+ \int_{N}F' \biggl(\frac{\Vert u^{*}h \Vert ^{2}}{4} \biggr)2 \lambda_{\alpha }\lambda_{m} h \bigl(u _{i}^{\alpha } \epsilon_{\alpha },du(e_{j}) \bigr)h \bigl(du(e_{i}),du(e_{j}) \bigr)\,dv _{g} \\ &\quad \leq \int_{N}F'' \biggl(\frac{\Vert u^{*}h \Vert ^{2}}{4} \biggr) \lambda_{\alpha } \lambda_{\beta }h \bigl(u_{i}^{\alpha } \epsilon_{\alpha },\sigma_{u}(e_{i}) \bigr)h \bigl(u _{j}^{\alpha }\epsilon_{\alpha },\sigma_{u}(e_{j}) \bigr)\,dv_{g} \\ &\quad \quad {}+ \int_{N}F' \biggl(\frac{\Vert u^{*}h \Vert ^{2}}{4} \biggr) \biggl[2 \lambda_{\alpha }^{2}+2 \lambda_{\alpha } \lambda_{m}- \biggl(\sum_{\beta } \lambda_{\beta } \biggr) \lambda_{\alpha } \biggr]h \bigl(u_{i}^{\alpha } \epsilon_{\alpha }, \sigma_{u}(e _{i}) \bigr) \,dv_{g}. \end{aligned}$$

If $F''(t)=F'(t)$, then (39) leads to the following inequality:

40 $$\begin{aligned} \sum_{A}I(V_{A},V_{A})& \leq \int_{N}F' \biggl(\frac{\Vert u^{*}h \Vert ^{2}}{4} \biggr) \bigl\Vert u^{*}h \bigr\Vert ^{2} \biggl\{ \bigl\Vert u^{*}h \bigr\Vert ^{2} \lambda_{m}^{2} \\ & \quad {}+ \max_{1\leq \alpha \leq m} \biggl[2\lambda_{\alpha }^{2}+2 \lambda_{\alpha }\lambda_{m}- \biggl(\sum _{\beta }\lambda_{\beta } \biggr) \lambda_{\alpha } \biggr] \biggr\} \,dv_{g} . \end{aligned}$$

If there exists a constant $c_{F}$ such that $\frac{F'(t)}{t^{c_{F}}}$ is nonincreasing, it follows that $F''(t)t\leq c_{F}F'(t)$ on $t\in (0,\infty )$, thus (39) implies

41 $$\begin{aligned} \sum_{A}I(V_{A},V_{A})&\leq \int_{M}F' \biggl(\frac{\Vert u^{*}h \Vert ^{2}}{4} \biggr) \bigl\Vert u^{*}h \bigr\Vert ^{2} \biggl\{ 4c_{F} \lambda_{m}^{2} \\ & \quad {}+\max_{1\leq \alpha \leq m} \biggl\{ \biggl[2 \lambda_{\alpha }+2 \lambda_{m}- \biggl(\sum_{\beta } \lambda_{\beta } \biggr) \biggr] \lambda_{\alpha } \biggr\} \biggr\} \,dv _{g} . \end{aligned}$$

Now, if $u:N\rightarrow M^{m}$ is a nonconstant *F*-stationary map and (6) or (7) holds, then, from (41) or (40), we know that $\sum_{A}I(V_{A},V_{A})<0$ and *u* is unstable. □

### Corollary 4.2

*Let* $u:N\rightarrow S^{m}$ *be a nonconstant* *F*-*stationary map with* $m>4$, *where* *N* *is any compact Riemannian manifold*. *If* $c_{F}< \frac{m}{4}-1$ *or* $\Vert u^{*}h \Vert ^{2}< m-4$, *then* *u* *is unstable*.

## Conclusions

In this paper, we investigate *F*-stationary maps between the compact convex hypersurface $M^{m}$ and any compact Riemannian manifold *N*. Assume that the principal curvatures $\lambda_{i}$ of $M^{m}$ satisfy $0<\lambda_{1}\leq \cdots \leq \lambda_{m}$ and $3\lambda_{m}< \sum_{i=1}^{m-1}\lambda_{i}$, then every nonconstant *F*-stationary map from $M^{m}$ to *N* or from *N* to $M^{m}$ is unstable if (6) or (7) holds. We mainly use the second variation formula for *F*-stationary maps (cf. [5]) to get the instability. In particular, we consider $S^{m}$ as a special case of compact convex hypersurfaces and obtain similar inferences.
